# Cytotoxic agents are detrimental to bone formed by distraction osteogenesis

**DOI:** 10.1007/s11751-013-0179-2

**Published:** 2013-10-09

**Authors:** Fergal P. Monsell, James Ralph Barnes, M. C. Bellemore, L. Biston, Allen Goodship

**Affiliations:** 1 Department of Paediatric Orthopaedic Surgery, Bristol Royal Hospital for Children, Paul O’Gorman Building, Upper Maudlin Street, Bristol, BS2 8BJ UK; 2 Department of Orthopaedic Surgery, Royal Alexandra Hospital for Children, Sydney, Australia; 3 Prince of Wales Medical Research Institute and Faculty of Medicine, University of New South Wales, Sydney, Australia; 4 Institute of Orthopaedics and Musculoskeletal Science UCL, Royal National Orthopaedic Hospital, Stanmorem, London, UK

**Keywords:** Osteogenesis, External fixator, Cytotoxic, Chemotherapy, Rabbit, Tibia, Bone tumor

## Abstract

Distraction osteogenesis can be used to replace segmental bone loss when treating malignant bone tumors in children and adolescents. These patients often receive cytotoxic chemotherapy as part of their treatment regimen. The effect of cytotoxic drugs on the cellular processes during distraction osteogenesis and the structural and mechanical properties of regenerate bone is unknown. We therefore used a rabbit model of distraction osteogenesis to determine that cytotoxic agents had a detrimental effect on regenerate bone formed by this technique. We administered adriamycin and cisplatinum to 20 rabbits using two different simulated chemotherapy regimens. All rabbits underwent an osteotomy at 12 weeks of age. Distraction osteogenesis began 24 h later at a rate of 0.75 mm a day for 10 days, followed by 18 days without correction to allow for consolidation. Regenerate bone was assessed using plain radiographs, bone densitometry, and mechanical testing. Peri-operative chemotherapy decreased the mechanical properties of the regenerate with regard to yield strain (3.7 × 10^−2^ vs. 5.2 × 10^−2^) and energy at yield (2.73 × 10^7^ vs. 3.92 × 10^7^). Preoperative chemotherapy in isolation reduced bone mineral density (0.38 vs. 0.5 g/cm^2^), bone mineral content (0.24 vs. 0.36 g), and volumetric bone mineral density (0.57 vs. 0.65 g/cm^2^) with no alterations in the mechanical properties. Conclusions: Preoperative chemotherapy appears to decrease the volume of regenerate bone, without affecting structural integrity, suggesting that the callus formed is of good quality. The converse appears true for peri-operative chemotherapy.

## Introduction

The treatment of malignant bone tumors in children and adolescents has improved over the last four decades as a result of progress in many disciplines. The development of potent cytotoxic agents has been of fundamental importance and initially involved a multiagent approach [8, 17, 27, 28]. Subsequently, a regimen using only two agents (adriamycin and cisplatinum) not only produced equivalent 15-year disease-free survival (DFS) and overall survival (OS) [4, 35], but that there was also only a 9 % rate of local recurrence [34]. This decreased rate of local recurrence has allowed limb-sparing surgery to be used more frequently after tumor excision.

The use of endoprostheses in the reconstruction of the distal femur has been reported to give good or excellent functional results in 83 % of patients, with a 10 year survivorship of 77 % [3, 26]. Even in patients with substantial growth remaining, expandable and adjustable prostheses have shown satisfactory functional outcomes in 71 % of patients, assessed using the American Musculoskeletal Tumor Society (AMSTS) functional rating system [6, 10, 32].

Reconstruction using extensive allograft has also been described for treating osteogenic and Ewing’s sarcoma but is associated with 77 % complication rate (other than a limb-length discrepancy) and 54 % rate of allograft fracture [1, 20]. Reconstruction with microvascular-free fibular grafts has also been reported with decreased fatigue fracture and nonunion compared to allograft and an AMSTS of good or excellent in 85 % of patients [11, 23, 33].

Distraction osteogenesis is a surgical technique sometimes used to regenerate bone, equalize limb lengths, and replace segmental bone loss [5, 12–15]. Tsuchiya et al. [37] also reported a decreased external fixation index (34 days/cm vs. 40 days/cm) and good or excellent functional results in 89 % of patients when using this technique compared to bone transport when reconstructing extensive defects (average 8.4 cm) after excising skeletal tumors [36]. Distraction osteogenesis relies on the ability of the body to generate enough bone to not only fill the defect left by tumor excision, but also to be robust enough to allow the individual to weight bear. Any interruption to these cellular processes could have a detrimental effect on new bone formation, which in turn could lead to failure through the regenerate bone. To date, we do not know the effect of a multiagent chemotherapy regimen on these processes, and this study was designed to address the deficiencies in the literature.

The questions we aimed to address in our study were as follows:

1. Do cytotoxic agents affect the structural properties of new bone formed by distraction osteogenesis?
2. Do cytotoxic agents affect the mechanical integrity of regenerate bone?
3. Does the timing of chemotherapy influences this affect?

## Materials and methods

Forty rabbits were divided equally and assigned on a random basis into two groups. Preoperative cycles of cisplatinum and adriamycin were used to simulate the clinical protocols reported by Malawer et al. [19] and pre- and postoperative cycles to simulate the cytotoxic regimen used by the European Osteosarcoma Intergroup [4, 35].

In the preoperative chemotherapy group, cisplatinum and adriamycin were administered in combination at 8 weeks of age, with a second infusion 14 days later at 10 weeks of age.

In the postoperative group, cisplatinum and adriamycin were administered in combination 10 days prior to and 4 days after the osteotomy, in order to standardize the interval between infusions at 14 days.

**Fig. 1:**
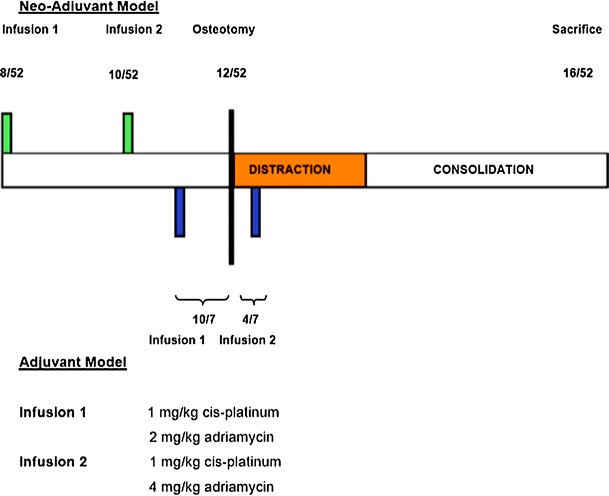
Illustration showing the chemotherapy regimens used

In both groups, the infusion was either an initial 1 mg/kg cisplatinum and 2 mg/kg adriamycin followed by a second infusion of 1 mg/kg cisplatinum and 4 mg/kg adriamycin or an identical volume of normal saline at identical points (Fig. 1).

We did not perform a priori power analysis as the literature was deficient, and no relevant data were available. This study should be regarded as a pilot and has generated an hypothesis that will form the basis of further research.

For all surgical procedures, animals were premedicated 30 min before with 10 mg/kg intramuscular ketamine (Parnell Laboratories Australia Pty Ltd, Alexandria, New South Wales, Australia), 4 mg/kg of xylazine (Ilium Xylazil-20; Troy Laboratories Pty Ltd, Smithfield, New South Wales, Australia), and 0.05 mg/kg of buprenorphine (Reckitt & Coleman Products Ltd, Hull, UK). General anesthesia was induced through a face mask using an enflurane (Abbott Australia Pty Ltd Botany, New South Wales, Australia) and a nitrous oxide/oxygen mixture. After each anesthetic, the animal was observed until alert and drinking before being returned to its cage.

Through an incision in the right groin, a 20-gauge intravenous cannula (Terumo Corporation, Tokyo, Japan) was inserted into the femoral vein and secured between two silk sutures providing a watertight seal. About 50 mL adriamycin/cisplatinum (intervention) or 50 mL normal saline (control) was infused over 2 h using an IVAC^®^ syringe pump (ALARIS Medical Systems Australia, Pty Ltd Seven Hills, New South Wales, Australia). Two weeks later, a second infusion was administered using an identical approach through the left femoral vein.

The tibial osteotomy was performed at 12 weeks of age in an attempt to reproduce the stage of skeletal development equivalent to a human adolescent [16]. The right leg was shaved and prepared with chlorhexidine. A medial longitudinal skin incision was used with subperiosteal exposure of the tibia along its length. Three-millimeter external fixator pins (Orthofix, Bussolengo, Italy) were inserted 1.5 cm proximal and distal to the midtibial diaphysis using a standard jig and power drill. We determined the positions of the remaining two pins using the jig to create a standard configuration of pins accurately aligned along the length of the tibia. An M100 monolateral fixator (Orthofix) was then secured approximately 1.5 cm from the skin to accommodate for postoperative swelling. The osteotomy was performed by predrilling the diaphysis and completing the osteotomy with bone cutters.

**Fig. 2:**
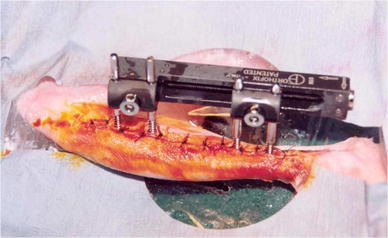
Orthofix *M* 100 External fixator in situ after middiaphyseal tibial osteotomy

The wound was then closed with interrupted sutures (Fig. 2) and dressed with povidone-iodine ointment (Professional Disposables Inc, Orangeburg, NY, USA).

Distraction osteogenesis began 24 h after surgery. The fixator was distracted by a half turn every 12 h for 10 days, followed by a period of 18 days to allow the consolidation of the regenerate.

**Fig. 3:**
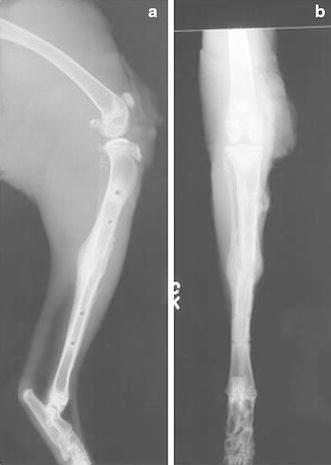
**a** Lateral and **b** AP radiographs of the tibia after removal of the fixator. Note the elliptical callus formation, the limb alignment, and the distance between the osteotomy and the pin sites

Animals in both arms of the study were euthanized. At 16 weeks of age, the right hind limb was disarticulated and aligned to produce consistent craniocaudal and mediolateral images with a Siemens (Erlangen, Germany) Multix H/UPH configuration with a focus to film distance of 100 cm and a 50-kV (±2 mV) and 4-mA exposure (Fig. 3). A single observer (FPM) assessed regenerate length, AP, and lateral alignment on the orthogonal images.

Bone density measurements were made using a total body dual-energy X-ray densitometer (DXA; LUNAR DPX; LUNAR Radiation Corp, Madison, WI, USA) and software designed for measuring small animals (LUNAR DPX, Small Animal Software Version 1.0; LUNAR Radiation Corp). The distance from the knee to the top of the callus and the height of the callus were measured and recorded. Each scan produced values for bone mineral density (BMD), bone mineral content (BMC), and volumetric bone mineral density (vBMD).vBMD was calculated assuming that the bone is an elliptical cylinder.

$$\text{The}\;\text{area}\;\text{of}\;\text{the}\;\text{cross - section}\;\text{of}\;\text{bone}=\pi \times \underset{2}{\left(\text{AP}\;\text{Bone}\;\text{Width}\right)}\times \underset{2}{\text{(Lat}\;\text{Bone}\;\text{Width)}}.$$

The volume of the region V = Cross-sectional Bone Area × ROI Height

$$\text{Volumetric}\;\text{BMD}\left(\text{g}/{\text{cm}}^{3}\right)\;=\;\underset{\text{V}}{\text{BMC}}$$

The tibiofibular complex was embedded in the mounting block and placed in an Instron mechanical testing machine (Instron Corporation, Norwood, MA, USA). Similar to comparable studies [18], the tibia was loaded in compression to failure at a rate of 2 mm/min using a 10-kN load cell. The length of exposed bone (lo) was measured before compression testing. This was standardized during mounting, ensuring that the polyester resin covered the screw holes adjacent to the regenerate. It was assumed that the regenerate was elliptical, and the mean area was calculated from DXA measurements. The load/displacement data were transformed to produce stress/strain data using the following formula:

$$\frac{\text{Stress}}{\text{Strain}}=\frac{\text{Load}/\text{Area}}{\text{Displacement}/{\text{l}}_{\text{o}}}$$

The data were transferred to Easy Plot (Spiral Software, Maryland, USA), and stress strain curves were produced for each specimen.

**Fig. 4:**
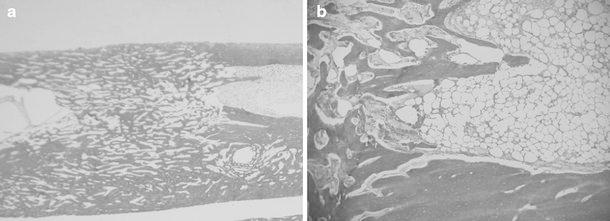
**a** Hematoxylin and eosin stain (original magnification, × 4) showing consolidated regenerate bone at the tibial osteotomy site after distraction. **b** Hematoxylin and eosin (original magnification, × 10) showing medullary/regenerate interface showing partial modularization of the regenerate bone

After mechanical testing, the regenerates were decalcified and stained with hematoxylin and eosin. One assessor (JRB) ( ) blinded to the identity of each specimen was used to undertake histological examination of each slide (Fig. 4a, b). Cortical thickening was assessed by taking [3] separate measurements from the endosteal to the periosteal surface of the regenerate bone, and the mean thickness was calculated for each specimen and used for comparison. Modularization was determined by the trabecular bone volume. This is the relative volume of total cancellous bone occupied by trabeculae, expressed as a percentage [25]. A Shapiro–Wilk test was used to confirm data produced by mechanical testing, and DEXA analysis was normally distributed, before an unpaired *t* test was used. The two-sided statistic with a 95 % confidence interval was used in each case.

For histological analysis, a Mann–Whitney *U* test was used to compare the histological parameters between intervention and control groups.

## Results

Structurally, preoperative chemotherapy had a detrimental effect on BMD (*p* < 0.01), BMC (*p* = 0.02), and vBMD (*p* = 0.04; Table 1, data produced by DEXA analysis) compared with a control population.

**Table 1:** Data obtained from DXA

	Pre-operative group	Peri-operative group
Mean value of BMC (g)		
Intervention	0.24	0.23
Control	0.36	0.22
*p* value	0.02	0.79
Mean value of BMD (g/cm2)		
Intervention	0.38	0.4
Control	0.5	0.4
*p* value	<0.01	0.97
Mean value of vBMD (g/cm3)		
Intervention	0.57	0.67
Control	0.65	0.63
*p* value	0.04	0.34

We observed a reduction (*p* = 0.97) in the modulus of elasticity from 5.47 to 5.15 (×10^3^), which would be expected, because the modulus/stiffness is dependent on the degree of mineralization (Table 2).

**Table 2:** Data obtained from mehanical testing

	Pre-operative group	Peri-operative group
Mean modulus of elasticity (Pa)		
Intervention	5.88 × 10^8^	5.65 × 10^8^
Control	5.92 × 10^8^	4.62 × 10^8^
*p* value	0.97	0.32
Mean energy at yield		
Intervention	2.45 × 10^7^	2.73 × 10^7^
Control	3.14 × 10^7^	3.92 × 10^7^
*p* value	0.31	0.01
Mean yield stress		
Intervention	1.64 × 10^7^	1.68 × 10^7^
Control	1.63 × 10^7^	1.89 × 10^7^
*p* value	0.94	0.39
Mean yield strain		
Intervention	3.2 × 10^−2^	3.7 × 10^−2^
Control	3.7 × 10^−2^	5.2 × 10^−2^
*p* value	0.44	0.01
Mean energy at failure		
Intervention	4.92 × 10^7^	5.25 × 10^7^
Control	5.48 × 10^7^	6.41 × 10^7^
*p* value	0.59	0.27
Mean failure stress		
Intervention	1.95 × 10^7^	1.89 × 10^7^
Control	1.87 × 10^7^	1.98 × 10
*p* value	0.71	0.67
Mean failure strain		
Intervention	4.7 × 10^−2^	5.2 × 10^−2^
Control	5.4 × 10^−2^	6.4 × 10^−2^
*p* value	0.41	0.11
Mean postyield energy		
Intervention	5.63 × 10^7^	2.51 × 10^7^
Control	2.34 × 10^7^	2.48 × 10^7^
*p* value	0.31	0.97

There was no difference in BMD, BMC, or vBMD in the peri-operative group compared with the control. Histologically, there was a trend for decreased cortical thickness in both chemotherapy groups compared with control populations, but these data were not statistically significant (*p* = 0.06 and 0.12; Table 3, data produced by histological assessment).

**Table 3:** Histologiocal data

	Mean cortical thickness (mm)
Peri-operative chemotherapy	
Intervention	2.51
Control	3.81
*p* value	0.06
Postoperative chemotherapy	
Intervention	2.46
Control	3.12
*p* value	0.12

With regard to mechanical properties, there was a major difference in the average yield strain and energy at yield of the peri-operative group when compared to the control population (Table 2, data produced by mechanical testing).

## Discussion

The aim of this study was to investigate the effects of multiagent cytotoxic agents on both the mechanical and structural properties of regenerate bone formed by distraction osteogenesis in a rabbit model. The clinical relevance of this work was to highlight the concerns with reconstruction of segmental defects after malignant bone tumor excision by distraction osteogenesis in the presence of cytotoxic chemotherapy. But the limitations of the study are clear and direct comparisons should not be made.

Interpretation of the clinical relevance of this work requires an analysis of the effect on the material and mechanical characteristics of the regenerate bone. The clinical issue is whether observed differences matter and are of sufficient concern to prevent reconstruction by this method.

Readers should be aware of the limitations of our study. First, the group sizes proved to be barely adequate to allow robust statistical analysis of the mechanical parameters, and future work should focus on a single specific area with a larger number of animal pairs. Second, we used plain radiographs to assess the morphology of the regenerate and determine the alignment of the lengthened bone in two orthogonal (AP and lateral) planes; however, the accuracy of plain radiographs is limited. Third, DXA does not account for the spatial distribution and inherent material properties of the tissues. While DXA has been used in similar studies 18, alternative methods of assessment include peripheral quantified CT (pQCT) and single photon emission CT. pQCT would have offered a potentially superior method of assessment [21, 22] and should be considered as a method of assessment in future work. Fourth, the regimen chosen was rudimentary when compared to current multiagent regimens used in clinical practice. The purpose of this study was to investigate the effects of cytotoxic agents on osteogenesis in general and not the efficacy of a specific regimen.

Decrease in weight has been identified in previous animal studies as an indicator of cytotoxic effect [38]. This effect is also seen in human clinical practice and is the result of several factors, including anorexia and a catabolic response to cytotoxic drugs. We demonstrated this difference in each experimental group, suggesting that the animals received clinically relevant amounts of cisplatinum and adriamycin. It was not possible to quantify the mechanism leading to this observation, because food and water were given ad libitum.

There were major differences noted in BMC, BMD, and vBMD with reduction in the preoperative chemotherapy group. This observation was likely to be a real effect and contradicts previous work [7] that did not demonstrate an alteration in DXA parameters after cisplatinum infusion. A possible explanation is that the addition of adriamycin has produced a more profound effect on regenerate mineralization, but more detailed comment is not possible from the data produced.

Failure occurs on the compression side of an eccentrically loaded bone, leading to progressive bending of regenerate, which is observed in clinical practice. This mode of failure is simulated by axial compression, which was considered most representative of the in vivo mode of failure. Previous studies [2, 24] have demonstrated a relationship between structural and mechanical properties of bone with this type of compression testing, and a simple uniaxial loading test provided a reproducible method of analysis for this experiment.

The experiment required reproducible longitudinal alignment of the bone during compression testing, and this was achieved using a mounting block. The mounting block consisted of two cylinders with an adjustable connector to accommodate variable bone lengths.

The lack of effect on the structural properties of regenerate is in agreement with Gravel et al. [9], even with the addition of cisplatinum.

The mechanism in which adriamycin and cisplatinum affect regenerate mineralization is not clear. The formation of regenerate bone involves a sequence of events, including neoangiogenesis [29], chondrocyte differentiation with expression of bone matrix proteins [31], and collagen I formation [39]. Adriamycin causes structural alterations in normal bone with reduction in cortical thickness, histological changes within the physeal plate, marrow hypoplasia [40], and specifically inhibits prolyl hydroxylation, leading to loss of stability of procollagen [30]. Cisplatinum causes histomorphometric changes in bone, including decreased volume of mineralized bone volume and decreased proportion of osteoblast-covered bone [7].

In the peri-operative group, there was no observed difference in bone mineralization, which concurs with previous work by Erhart et al. [7]. The power of these observations was however low, and this may represent a Type II error. The high power of the equivalent observations in the preoperative group is highly suggestive of a real effect, and the difference between groups may be spurious.

Of interest in the peri-operative group are the major differences in energy at yield and yield strain with difference in means of 30 % (energy at yield) and 29 % (yield strain) and appropriate statistical power. This is suggestive of a real effect and may have clinical implications, particularly because the main concern in human practice is of regenerate failure after fixator removal. The reduction in energy at yield after cytotoxic chemotherapy is likely to be clinically important and if translated into human practice may represent an increased risk of regenerate failure after limb reconstruction. This may require either prolonged fixator wear or external support after fixator removal; however, further research is required to quantify this. Definition of a chemotherapy regimen that would have a clinically irrelevant effect on the material properties of the regenerate is beyond the scope of this experiment.

It has been possible to develop a reproducible model of limb lengthening in this species. Bone is consistently formed in the distraction gap, and the overall alignment was satisfactory in all lengthened limbs. This model is suitable for future experiments investigating limb lengthening in the juvenile New Zealand white rabbit. We have observed that cytotoxic chemotherapy has a detrimental affect on bone formed in a lapine model of distraction osteogenesis. This should add a note of caution to the use of this technique for reconstruction in a clinical context, but this study is unable to address the effect of conventional chemotherapy regimens in human subjects.
